# Supplementary material for: Photovoltage-Driven Photoconductor Based on Horizontal p-n-p Junction
Source: Nanomaterials (Basel). 2024 Sep 12;14(18):1483. doi: 10.3390/nano14181483 (PMC11435119; doi:10.3390/nano14181483)
Supplement: Supplementary file 1 [file nanomaterials-14-01483-s001.zip › nanomaterials-3188401-supplementary.pdf]

Supporting Information

Photovoltage-Driven Photoconductor Based on Horizontal *p-n-p* Junction

Feng Han [1], Guanyu Mi [2], Ying Luo [2,*] and Jian Lv [2,*]

[1] School of Defence Science&Technology, Xi'an Technological University, NO, 2 Xuefu Middle Road, Xi'an 710021, China; hanfeng202408@126.com

[2] School of Optoelectronic Science and Engineering, University of Electronic Science and Technology of China, Chengdu 610054, China; 202011050821@std.uestc.edu.cn

* Correspondence: yingluo907@uestc.edu.cn (Y.L.); lvjian@uestc.edu.cn (J.L.)

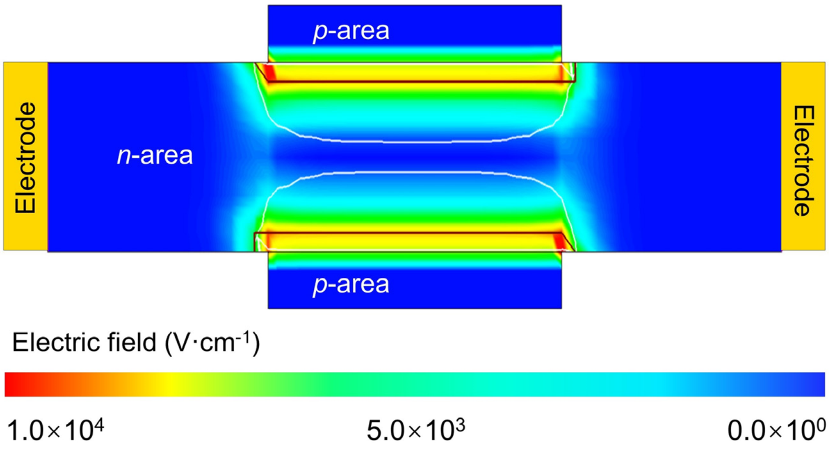

Figure S1. Simulated electric field of the PVPC.

According to the depletion layer width formula of the *p-n* junction[1] (eq (S1)), where W_n is the depletion layer width in the *n*-area, ε_0 is the vacuum dielectric constant, ε_{Ge} is the relative dielectric constant of Ge, q is the elementary charge, V_{bi} is the built-in field, k_B is the Boltzmann constant, T is the temperature, N_a is the acceptor concentration, N_d is the donor concentration, and n_i is the intrinsic carrier concentration of Ge, the increase in doping concentration causes W_n to decrease (Figure S2). A narrow depletion layer width is not conducive to suppressing dark current.

$$W_n = \left\{\frac{2\varepsilon_0\varepsilon_{Ge}V_{bi}}{q}\left[\frac{N_a}{N_d}\right]\left[\frac{1}{N_a+N_d}\right]\right\}^{\frac{1}{2}} = \left\{\frac{2\varepsilon_0\varepsilon_{Ge}}{q}\frac{k_BT}{q}ln\left(\frac{N_aN_d}{n_i^2}\right)\left[\frac{N_a}{N_d}\right]\left[\frac{1}{N_a+N_d}\right]\right\}^{\frac{1}{2}} \quad \text{(S1)}$$

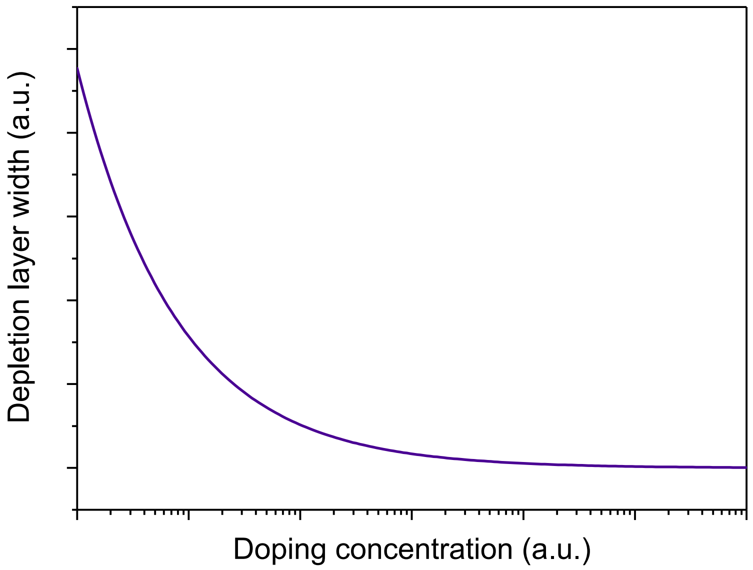

Figure S2. Calculated deletion layer width as a function of doping concentration.

Note S1. Photodetection capability of the PVPC

Given the fast response speed of the PVPC, it possesses a high bandwidth, enabling the use of shot noise as an approximation for its optimal noise scenario[2]. The formula for calculating shot noise (i_n) is $i_n = (2qI_d\Delta f)^{1/2}$, where q is the elementary charge, I_d is the dark current, and Δf is the bandwidth. The calculated shot noise for the PVPC is 5.36×10^{-14} A·Hz$^{-1/2}$. According to the formulas for equivalent noise power (NEP) and specific detectivity (D^*), $NEP = i_n/R$ and $D^* = (R/i_n)\times(A_d\Delta f)^{1/2}$, where R is the responsivity and A_d the photosensitive area, a NEP of 2.75×10^{-15} W·Hz$^{-1/2}$ and a D^* of 1.15×10^{11} cm·Hz$^{1/2}$·W^{-1} for the PVPC can be obtained.

References

1. S. M. Sze, Ng, K. K. , "Physics of Semiconductor Devices, 3rd Edition," 2007.
2. F. Wang, T. Zhang, R. Xie, Z. Wang and W. Hu, "How to characterize figures of merit of two-dimensional photodetectors," *Nat Commun*, vol. 14, no. 1, pp. 2224, 2023.
